# Microwave technology: a novel approach to the transformation of natural metabolites

**DOI:** 10.1186/s13020-021-00500-8

**Published:** 2021-09-16

**Authors:** Qi Hu, Yanan He, Fang Wang, Jing Wu, Zhimin Ci, Lumeng Chen, Runchun Xu, Ming Yang, Junzhi Lin, Li Han, Dingkun Zhang

**Affiliations:** 1 State Key Laboratory of Southwestern Chinese Medicine Resources, Pharmacy School, Chengdu University of Traditional Chinese Medicine, Chengdu, 611137 China; 2 State Key Laboratory of Innovation Medicine and High Efficiency and Energy Saving Pharmaceutical Equipment, Jiangxi University of Traditional Chinese Medicine, Nanchang, 330004 China; 3 Xinqi Microwave Co., Ltd., Guiyang, 550000 China; 4 TCM Regulating Metabolic Diseases Key Laboratory of Sichuan Province, Hospital of Chengdu University of Traditional Chinese Medicine, Chengdu, 610072 China

**Keywords:** Microwave heating, Extraction, Drying method, Metabolites, Structural change

## Abstract

Microwave technology is used throughout the world to generate heat using energy from the microwave range of the electromagnetic spectrum. It is characterized by uniform energy transfer, low energy consumption, and rapid heating which preserves much of the nutritional value in food products. Microwave technology is widely used to process food such as drying, because food and medicinal plants are the same organisms. Microwave technology is also used to process and extract parts of plants for medicinal purposes; however, the special principle of microwave radiation provide energy to reaction for transforming chemical components, creating a variety of compounds through oxidation, hydrolysis, rearrangement, esterification, condensation and other reactions that transform original components into new ones. In this paper, the principles, influencing factors of microwave technology, and the transformation of natural metabolites using microwave technology are reviewed, with an aim to provide a theoretical basis for the further study of microwave technology in the processing of medicinal materials.

## Introduction

Microwave technology is a modern technology that produces heat energy by the rapid movement and rotation of polar molecules by rubbing against each other. Microwave irradiation will induce thermal effects, electric field effects and other nonthermal effects in substances. The thermal effect may convert electromagnetic energy into thermal energy to generate heat. Nonthermal effects cause rotation and polarization of dipole molecules, which increases the frequency of collisions between molecules producing heat through friction and the heat transfer proceeds from the inside of the substance to the outside [1–3]. Microwave technology offers many advantages in the preparation of food: appearance, flavor, and nutritional composition of products heated by microwave radiation, at a level of quality that far exceeds conventional methods, while surface hardening of some products and other pitfalls can be avoided [4, 5]. In addition, the preservation of flavor can reduce the need for salt to maintain healthy levels in the human body [6]. While microwave technology has some drawbacks, conventional methods of cooking food change the texture, flavor, and appearance of most food and cause it to lose some or many nutrients in the process [7, 8]. Therefore, microwave technology is widely used in food preparation such as drying, baking, sterilization and thawing now [9], and has in turn received wide attention throughout the food industry.

Many edible plants also have medicinal properties. Therefore, microwave technology has also peaked interest in the pharmaceutical field. Studies have demonstrated how microwave technology can be used to dry plants, extract bioactive components, and sterilize herbs and medicinal plants [10–12]. And microwave heating can completely control the extraction time, power and other parameters [13]. In the process of research, scientists discovered that rapid heating and special principles of microwave radiation can reduce nutrition loss and maintain appearance, but the friction between mobilized molecules that generates the heat and the way in which selective heat acts on polar bonds during irradiation of the product, promoting certain compounds to form and chemical reactions, such as oxidation, dehydration, structural changes, and esterification reaction occur and transform secondary metabolites in medicinal plants to other structure.

In view of the unique properties of medicinal plants, it is important to know if the composition changes when plant material is dried using microwaves and if these changes are beneficial or harmful. In this paper, principle influencing factors including microwave oven and materials, and changes in metabolites caused by microwave radiation are presented and provide reference data for the use of microwave technology to process medicinal plants.

## Principles of microwave technology

**Fig. 1:**
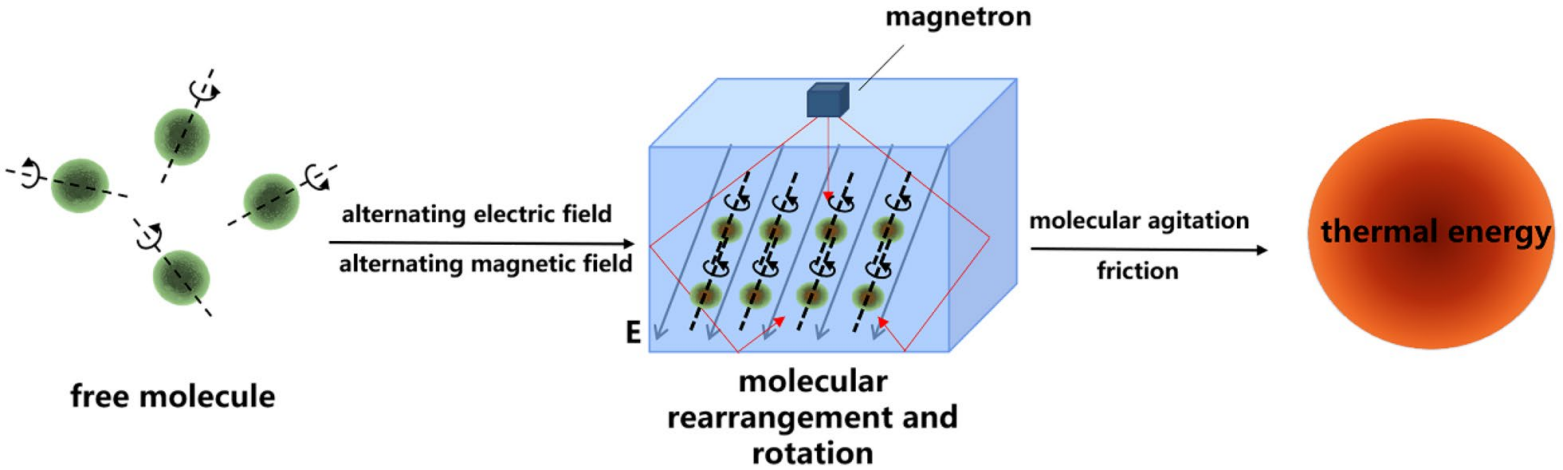
The principle of microwave technology

Microwaves do not generate heat, instead the heat is mainly generated by intermolecular frictional forces [14]. During microwave radiation, polar molecules present in food (e.g., water) change from state of disorder to an ordered structured aligned in a particular direction [9]. The structure changes as the frequency changes under the influence of an alternating electric field. The excited molecules move rapidly and the friction generated in these movements produce heat [15]. The principle of microwave technology is illustrated in Fig. 1. Studies have shown that polar molecules can vibrate 2.45 billion times per second under microwave radiation at frequency of 2450 MHz, so materials can be heated, dried, or cured in a short amount of time [16].

Heating with microwaves is often used to dry food. The operational principle is that the polarized molecules and frequent dipole interactions cause the water molecules to interact with other molecules and align in different directions under an electrical field. These forces cause the rate of internal water diffusion from the interior to the surface of the object to increase [17]. In addition, the mechanical action and thermal effect increase the fluidity of the water which further accelerates the motion [18].

**Fig. 2:**
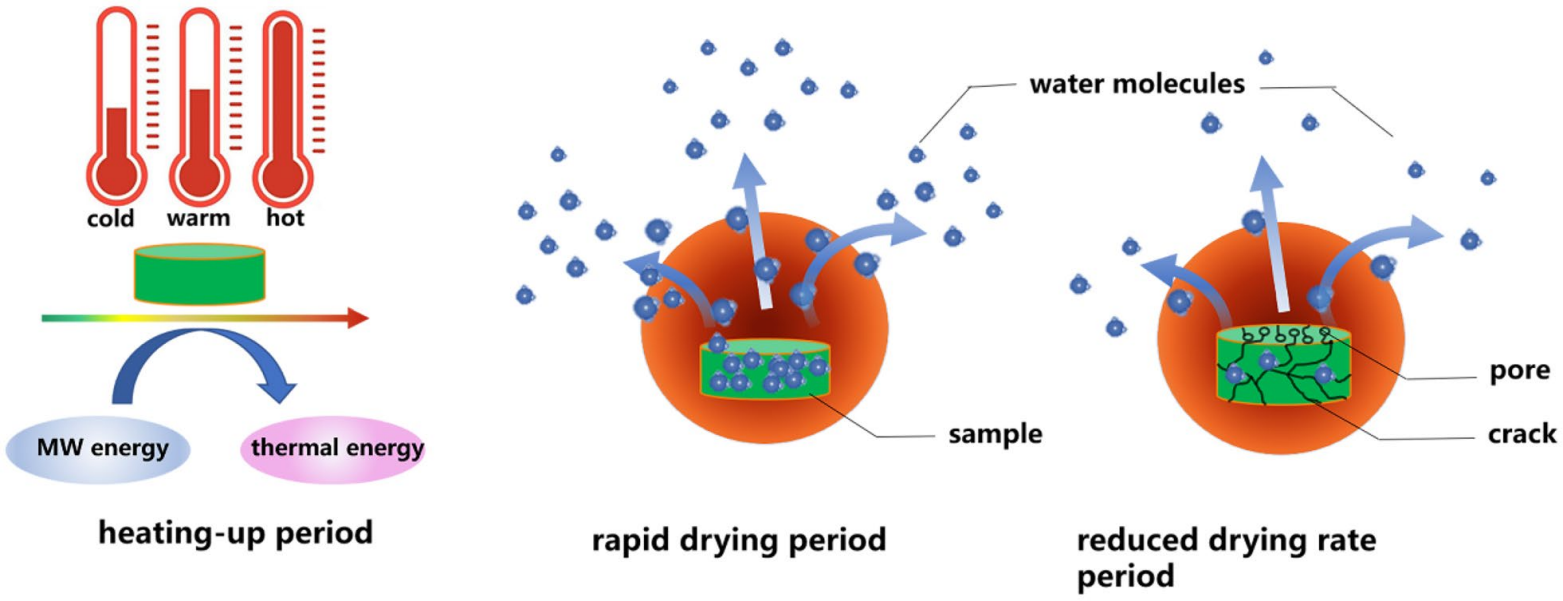
Three stages of drying material using microwave radiation

The effect of microwave radiation on water molecules also provide energy in the water helping it to maintain the indoor temperature, driving water from the interior of the materials to the surface [19]. The microwave drying process can be divided into three stages (Fig. 2) [20]. Microwave radiation can excite polar molecules steadily increasing movement, thus generating enough heat to transfer that thermal energy to the water in the material being heated; in this way plant material can be heated and dried, so as to achieve the purpose of heating and drying.

In the first phase, microwave energy is converted into heat. In the second phase, water evaporates rapidly through diffusion, and the third phase is a period of reduced rates of drying when the thermal energy exceeds the energy required to completely vaporize moisture.

## Factors influencing microwave technology

**Fig. 3:**
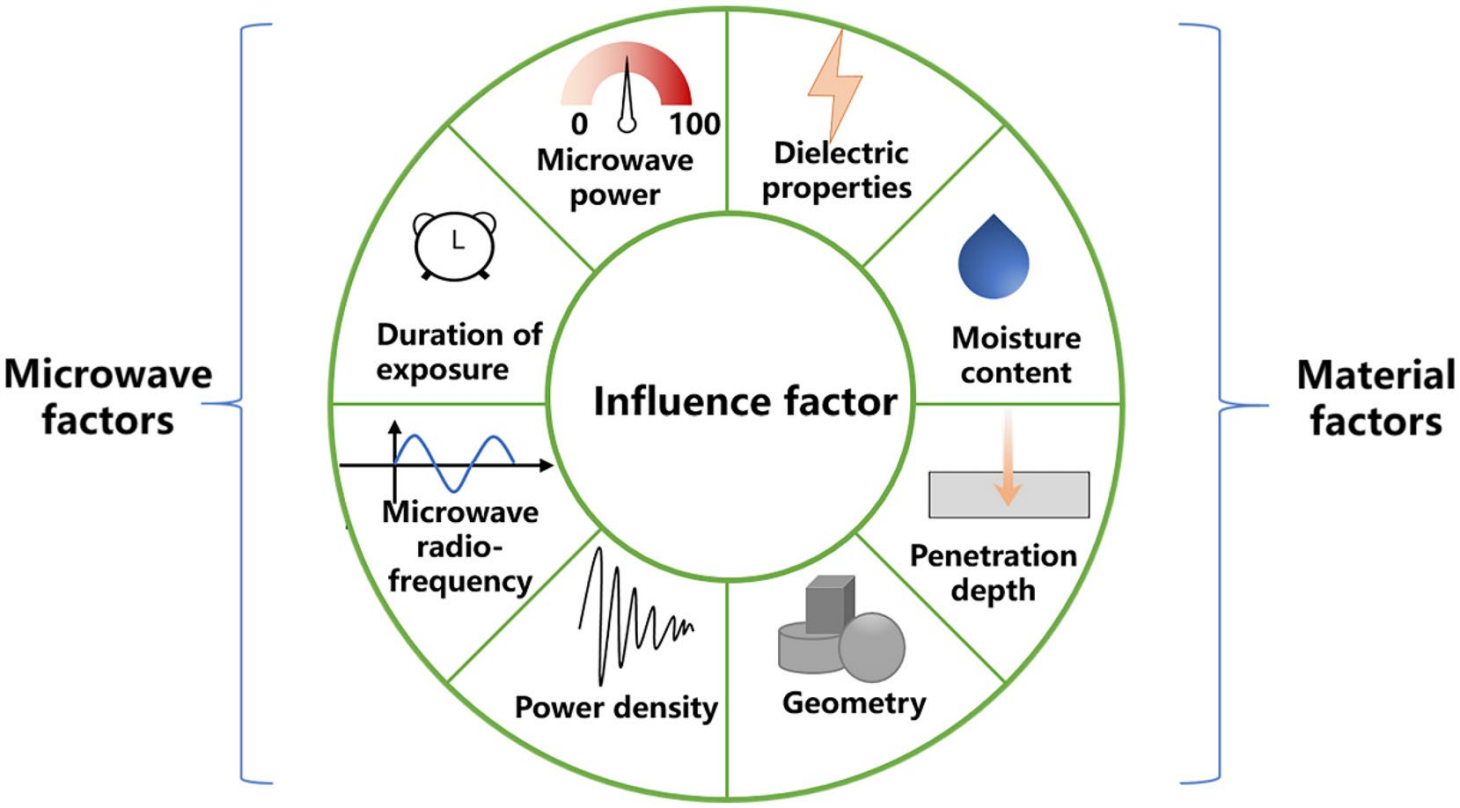
Influence factors of microwave technology

The heating effect of microwave technology is affected by a variety of factors, which can be divided into microwave factors and material factors. Microwave factors include microwave power, duration of exposures, radio frequency and power density. While material factors include dielectric properties, moisture, penetration depth and geometry (Fig. 3). Many factors interact with each other to affect the microwave heating effect.

### Microwave factors

#### Microwave power

The application of microwave radiation increased the isosteric heat or the adsorption energy of monomolecular layer of moisture, which increased the surface tension on the sample, thereby improving the absorption of moisture from the sample [21]. As advances in levels of power achieved, the degree at which a material breaks increased. Higher temperatures and power levels will damage the microstructure of materials to be dried [22]. Low power microwaves promote growth and reproduction in plants. After a short period of low power microwave radiation, the content of soluble protein increased and metabolism was activated in the roots of Sequoia plants [23]; however, if the power supply is too low, the structure of the material will also be damaged.

If there is not a suitable power supply, the temperature of the sample has little effective on moisture which may rise rapidly, causing damage to the sample’s structure [24]. After microwave heating, the effective component of the sample degrades, the microstructure changes and the volume expansion. Dehghannya et al. [17] found that when the power was 350 and 500 W, the microstructure of the apple changed greatly, which is attributed to a high rate of moisture diffusion. Compared to power level of 90 and 160 W, the internal structure of the sample damaged to a large extent; the volume density will decrease as well. The total phenolic content in tomato decreased significantly under high power microwave [25]. That is, high power microwaves will greatly affect the composition of natural products.

#### Duration of exposure

**Fig. 4:**
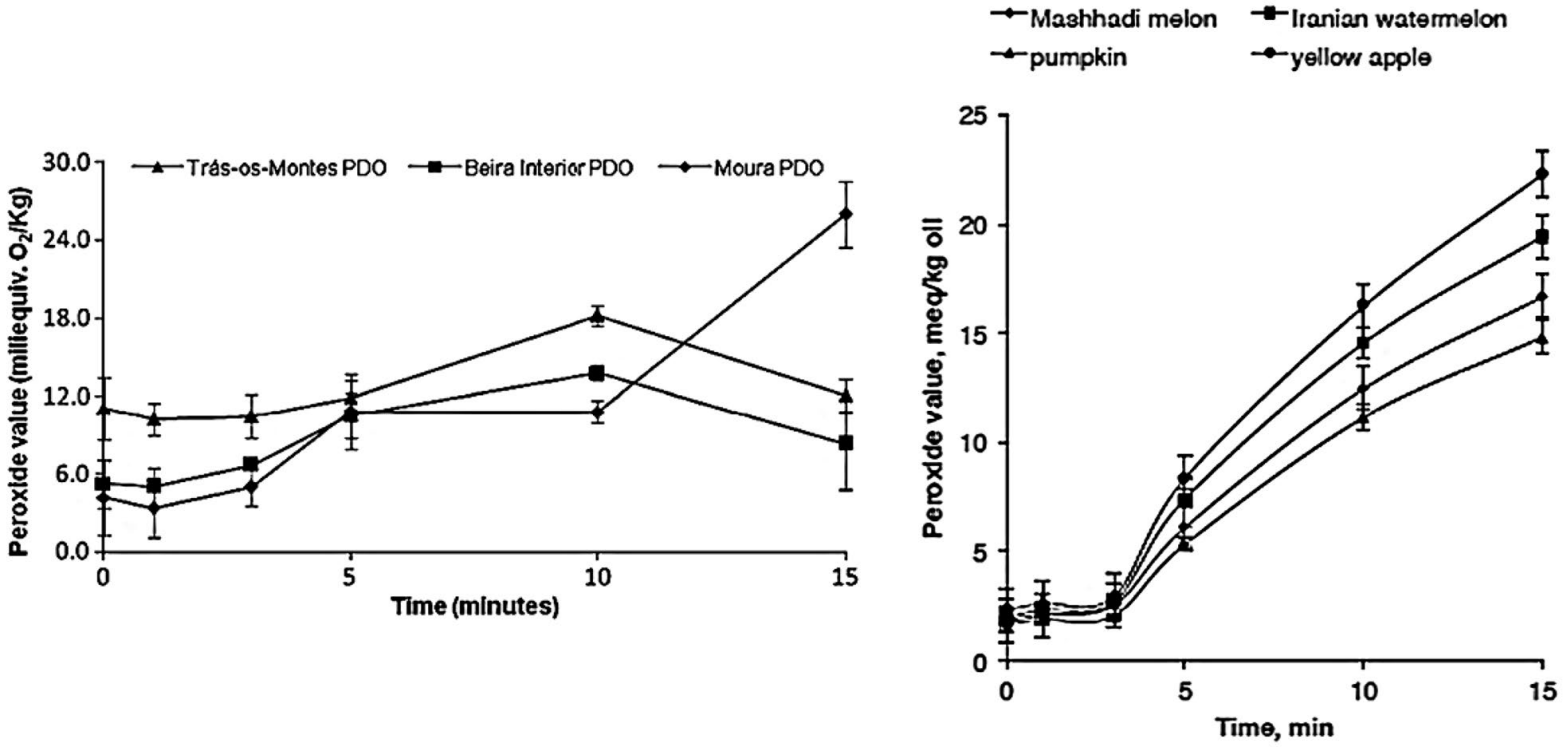
Lipid oxidation time (Figure used with permission from Elsevier, Copyright (2021) [28, 29])

The duration of microwave radiation will affect the properties of the heated materials. The longer the sample is exposed to microwave radiation, the more damage will be caused to the structure [26]. The greater the output power, the greater the influence of duration on the sample, and the functional components of the sample will quickly decompose [27]. Malheiro et al. [28] found that the longer the duration of heating, the more the content of chlorophyll and carotenoids in olive oil decreased. These changes were not obvious during the first 3 min, but the content decreased rapidly after 15 min of heating. Hashemi et al. [29] found that the degradation rate of the oil accelerated after microwave treatment for 15 min, longer exposure times caused the total phenolic content of the oil samples to experience loss. As shown in Fig. 4, the peroxide value of olive oil changed slightly in the first 3 min after microwave heating, but showed a significant upward trend from three minutes to ten minutes, and a sharp upward trend appeared after 10 min. For seed oil, the peroxide value hardly changed in the first 3 min of microwave heating, but showed an obvious upward trend after 3 min. Therefore, when heating with microwaves, they should be as short as possible. For different kinds of natural products, due to their different principal components, the appropriate time for microwave heating also varies. The appropriate time for different natural products is shown in Table 1. Vitamin cannot be exposed to long time microwave radiation due to their thermal sensitivity, while plant proteins can be exposed to long time microwave radiation due to their little effect of denaturation. Short-duration heating by microwave radiation can reduce degradation of components, and impart a protective effect on plant cells. One study showed that plant cells exposed to short intervals of microwave radiation were able to resist ultraviolet damage to the material [30].

**Table 1:** The optimal time for different natural products

Main components	Source	Optimal time	Reference
Polysaccharides	Eucheuma denticulatum	10 min	[31]
	*Ascophyllum nodosum*	15 min	[32]
Starch	Yam	5 min	[33]
	Potato	7 min	[34]
	Rice bran	3 min	[35]
	Corn	2–4 min	[36]
Lipids	Olive oil	3 min	[28]
	Seed oil	3 min	[29]
	Green gram seed	80 s	[37]
	Rapeseed	6 min	[38]
	Flax seed	6 min	[39]
Protein	Bean	9 min	[40]
	Chickpea	11 min	[40]
Vitamin	Broccoli	2 min	[41]
	Mallow	2 min	[41]
	Tomato	150–180 s	[42]

#### Microwave radio-frequency

The radio-frequency of microwaves also effect heating with microwave radiation. Commonly used frequencies are 915, 2450, and 5800 MHz, of which 2450 MHz is most commonly used in food processing [16]. Radio frequency also has an effect on the dielectric properties, and the degree of influence is related to temperature. At 20 °C, the radio frequency has a slight effect on the dielectric properties [43]. While at higher temperatures, the dielectric properties decreased significantly with increasing frequency. At higher temperatures, the permittivity decreased at higher frequencies significantly. At frequencies below 300 MHz, ionic conduction plays a dominant role in dielectric loss, decreasing the dielectric loss with increasing frequency.

#### Power density

Power density plays a key role in the microwave heating process. The moisture content effects heating of materials; the more content of free moisture in the materials, the more sensitive to microwave radiation [44]; however, during the heating process, the moisture content is reduced, power density correspond to moisture content can effectively improve the drying effect, so when moisture was detected and the power density of the microwave was adjusted timely, the drying process was improved. To explore the influence of power density on heating, Koné et al. [45] dried tomato samples using power density as the independent variable. The results showed that the quality of dried products was better when the power density was controlled, which may in turn controls the internal temperature of the product. Power density can be controlled by making adjustments to the microwave oven (e.g., reducing the magnetron output, increasing sample size, or increasing the size of the oven cavity [16]).

Because many of the microwave ovens are factory set, they are less liberal in accordance with mandated regulations; thus, factors such as radio frequency and power density cannot be controlled. Instead, we focused on the parameters that can be controlled, those related to the material to be heated by radiation, especially the changes in the geometry and moisture content of the material to optimize the heating effect.

### Material factors

#### Dielectric properties

In the process of heating with microwave radiation, the internal heat of the material is a function of its dielectric properties. Complex relative permittivity (∈ *) was used to characterize the dielectric properties of the material and its capacity to absorb microwave energy and store it as described in Eq. 1 [16]:

1 $$  \epsilon ^{*}  =  \epsilon ^{\prime}  - {\text{j}} \epsilon ^{\prime\prime}  $$

 ∈ ': dielectric constant; ∈ ": loss factor.

The dielectric properties of materials are related to temperature, frequency, purity, chemical state, and the heated process [9]. Dielectric properties can be improved by treating the material treatment and controlling conditions to optimize absorption of energy from the microwaves. This can be achieved by using ethanol instead of water as the solvent [46]. The main factors that affect dielectric properties include the frequencies comprising the alternating electrical field, moisture content, volume density, temperature, ionic properties, solution concentration, structure, and composition of the materials to be heated. Of these factors, volume density is considered to be the main factor that controls changes in the dielectric constant and rate of loss factor [47]. Effect of ionic properties is reflected in the increase of loss factor of salt solution [15]. Zhu et al. [43] used flour made from chestnuts as the research material; a network analyzer and an open-ended coaxial-line probe were used to measure the dielectric properties of the flour and found that the higher the dielectric loss factor, the greater the power absorbed by the material, and consequently, the rate of the temperature rise increases. The dielectric constant and loss factor decreased as frequency increased, and they increased as moisture content and temperature increased [43].

#### Moisture content

The effect of moisture content of materials on heating with microwave radiation is mainly determined by the dielectric properties of the material. In the range of microwave radiation, interactions between the electric field and biological tissue samples produce two kinds of dispersions, γ-dispersion and ionic conductivity. In plant tissues, γ-dispersion is determined by the orientation and induction of the dipolar molecules, while ionic conductivity represents the electrical conductivity of electrolytes in weak organic acids.

As the moisture content decreased, the value of the dielectric constant decreased [21]. It was seen from that the moisture content was affected by drying process in that the period required drying was reduced. When the moisture content of sample was high, the gas migrated from the interior was limited, thereby increasing the total pressure in the system and facilitating the transfer of heat through the material [48]. Li et al. [49] found that the higher the moisture content, the stronger the microwave effect of microwave radiation. Therefore, when using microwave heating, certain methods should be taken to seal the oven, to avoid rapid loss of moisture through evaporation, which decreased the heating effect of microwaves.

#### Penetration depth

The penetration depth of microwave energy affects the transfer of energy from the surface of the microwave oven to the interior [18]. If the penetration depth is less than the size of the sample, the energy will not be transferred completely to the sample, and so the absorption of energy by the product is inhibited, resulting in uneven heating. The penetration depth of many fresh foods is about 0.6–1 cm at 2450 MHz [16]. The penetration depth is negatively correlated with microwave frequency, moisture content and temperature [9]. Li et al. [50] discussed the factors influencing the uniformity of heating product with microwave energy; in terms of the material, the most important thing is to ensure that the depth of penetration is larger than the thickness of the material, results showed that heating was uniform and that a vertical rotary microwave performed better than static or horizontal rotary microwaves, proving that the penetration depth of the microwaves was different in different materials. When heating materials using microwaves, the material in the sample, can be made in different sizes to improve the speed of heating and the efficiency of energy utilization.

#### Geometry

Geometric shape influences the depth of penetration into the produce from variety of directions. A spherical shape is the ideal model because a sphere is uniform in every direction, while other geometric shapes cause nonuniform heating due to edge effects [51]. In terms of the amount of time required to heat the product to the desired temperature, smaller dimensions in thickness and volume attenuate energy more readily in relatively small or thin samples. For large volumes, more time is required to reach the boiling point and therefore more time to diffuse the energy thereby reducing the uniformity of temperature distribution. Using agar as an example, because the size of a plate of agar is smaller than the size of a sphere or cylinder, the time needed to heat the plate is shorter than other shapes, and the coldest point in each model occupies a central position of sample, which in turn transfers thermal energy from the surface of sample to its center [52]. Therefore, when considering the appropriate volume shape of the sample, the reason for heating it should be considered as well. When the distribution of temperature is not important, shapes with edges and corners are favored, and when uniform heating is needed, spherical shapes are preferable.

## Transformation of natural products

Primary metabolites and secondary metabolites are essential substances for plant growth and development, and are also the basis of their biological activities. Studies have shown that the metabolites will transform after microwave treatment, resulting in changes in taste, color, nutritional composition and biological activity of the natural products. It is helpful to improve the efficiency of microwave and obtain higher quality products by study the rule of metabolites transformation under microwave treatment.

### Primary metabolite

#### Polysaccharides

Polysaccharides are important bioactive ingredients that exhibit key biological functions with many protective properties. They are antioxidants, immunomodulatory factors, anti-tumor formation, and protect against hypoglycemic, hypolipidemic, and gastrointestinal conditions [53, 54]. After drying and extraction, the structure and physical and chemical properties of polysaccharides in natural products will be changed. However, the unique porous structure formed by microwave heating can make the polysaccharides show different properties. After treatment with microwaves, the viscosity, enzyme activity, content, and molecular weight of the polysaccharides change, which affects their functional characteristics.

In the process of microwave treatment, polysaccharides are susceptible because of their intensive polarity. And the porous structure formed by microwave radiation has a favorable effect on the hypoglycemia activity of polysaccharides. The chemical bond break caused by microwave can release more small molecule sugars and promote antioxidant activity. However, the reduced viscosity will affect its adhesion and affect the biological activity. For example, the anti-tumor activity of polysaccharides will be reduced after microwave treatment because of their low viscosity [55].

Hypoglycemia and the viscosity of soybeans and the solubility of polysaccharides increased, while α-amylase was inhibited after heating with microwaves. The structural analysis revealed that the surfaces of the polysaccharides were damaged, the surface area increased, the particles became smaller, and the functional radicals changed slightly [56]; however, the degradation of polysaccharides increased the glucose content. In addition, there are studies that show that the viscosity of polysaccharides decreased after treatment [57]. And likely hinders the hypoglycemic tendencies of polysaccharides.

*Eucommia ulmoides* Oliver leaf extract obtained using microwave technology changed the content, molecular weight, polydispersity, and molecular structure of the polysaccharides. Differences in carbohydrate content is caused by the breaking of hydrogen bonds between polysaccharides and other components in the cytoderm. The molecular weight and polydispersity of polysaccharides are high because microwave radiation promotes release of glucan. The antioxidant content increases as molecular weight increases [58]. The microwave-extracted ginseng polysaccharides extracted using microwaves has high antibacterial and antioxidant abilities, but as the power increases, the biological activity is destroyed due to the degradation of polysaccharides at high power [59]. However, several studies have shown that the smaller the molecular weight of polysaccharides, the higher the uronic acid content, and the better the antioxidant capacity [60–62]. After degradation, the molecular weight and viscosity of the water-extracted polysaccharides decreased, and the 2,2-diphenyl-1-picrylhydrazyl (DPPH) radical-scavenging activity increased as power increased [63].

Multiple studies have shown that microwaves influence bioactivity in polysaccharides, and their influence is related to their structure. Under normal circumstances, hydrogen bonds fracture easily because microwaves reduce viscosity in polysaccharide solutions. When carbohydrates of low molecular weight and viscosity caused by polysaccharide degradation are formed, the viscosity of the polysaccharide will increase. And the porosity in the structure caused by heating can increase hypoglycemic effects. Microwave has a greater impact on the strength of chemical bonds than on the physical structure of polysaccharides. Mechanisms of antioxidant activity in polysaccharides are not well understood, but it is generally believed that antioxidant activity in polysaccharides become stronger after degradation, but dextran, which is heavier than some polysaccharides exhibited strong antioxidant activity, and microwave treatment appears to facilitate the release of dextran. Therefore, the use of microwave treatments should be based on the internal structure of the polysaccharides and its functional properties.

#### Starch

Starch, a type of polysaccharide, is usually discussed separately. Compared with the biological activity of polysaccharides, people pay more attention to the digestibility of starch. While microwave treatment can obtain more slowly digestible starch, which is suitable for diabetic patients. The structural changes during microwave treatment can lead to gelatinization of starch and changes in its physical and chemical properties, which are the factors leading to changes in starch digestibility and stability.

**Fig. 5:**
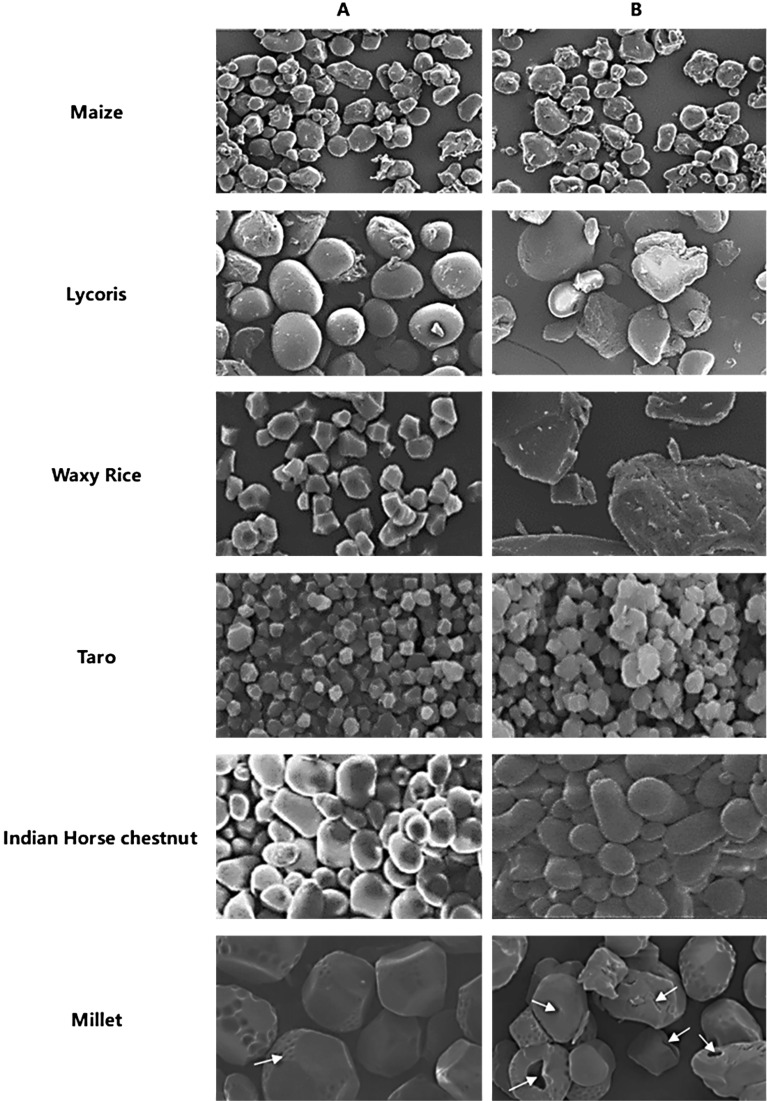
SEM of starch (**A**) Native (**B**) Microwave (Figure used with permission from Elsevier, Copyright (2021). [49, 64, 66–69])

After exposure to microwave radiation, polar molecules rotate quickly and generate heat by friction, causing the surface of starch particles to crack and shrink [14]. Polar radicals such as hydroxyls, carboxyls, and water molecules in starch vibrate rapidly, and destroy the double helix structure of starch, molecules as chemical bonds break and the enhancement of intermolecular force [49]. Specifically, the double helix structure of starch molecules is destroyed by the rapid vibration of the molecule. Different from the traditional heating, the selective effect of microwave heating on polar bonds can make the α-(1,6) glycosidic bond in the outer chain is destroyed specifically, and the broken starch chain is rearranged by the influence of microwave energy [64]. During rearrangement, starch molecules become polarized and the dielectric constant increases [65]. The broken amylopectin is rearranged into amylose [66]. Changes to the natural starch after treatment are shown in Table 2. As shown in Fig. 5, after microwave treatment, the starch not only expanded, but also appeared pores.

**Table 2:** Changes in the raw material (natural starch) after treating with microwave radiation

Source of raw material	Processing conditions	Changes observed in the starch	Reference
Maize	Moisture content up to 30% (w/w) 2450 MHz microwave oven Output power 1.2 kW	The content of amylopectin decreased, relative crystallinity increased initially then decrease, granules were completed, the degree of gelatinization, and digestibility increased	[64]
Chickpeas	2450 MHz microwave oven Power of 565 W (90% maximum power) Temperature 105 °C	Mineral content decreased slightly, starch gelatinized, slow digesting starch decreased, fast digesting starch increased	[40]
Lycoris	Heated for 120 s at a power level of 200 W, followed by heating at 50 °C in a convection oven until the moisture content was approximately 8%	Granules collapsed and developed the characteristics of a massive, rough or irregular surface; the tendency to aggregate was strong and retrogradation was poor	[67]
Rice	Power values of 4, 8, or 10 W/g for 3 min	The number of microcrystalline and periodic amorphous structures increased and digestibility decreased	[68]
Taro	The moisture content was adjusted to 25%, heated for 5 min at power level 180 W	Amylose content increase, the swelling and solubility of the modified starch increased, the water retention, and final viscosity increased	[66]
Indian Horse chestnut	Moisture content tempered to 22% ± 3% Exposure to microwave radiation for 15, 30 and 45 s	The morphology of starch granules was round and ellipse, the surface was smooth, the water absorption and light transmittance increased, the apparent amylose content decreased, and the antioxidant activity increased	[69]
Potato	Heated for 5 min in a laboratory microwave oven (2450 MHz) at 440 or 800 W	Viscosity decreases, heat treatment temperature is low, free radicals increase	[70]
Millet	Duration of exposure to microwave radiation was 60 s The power level was 700 W, and the moisture content was adjusted to 30%, 35%, 40%, 45%, and 50%	The peak viscosity decreased, the swelling force decreased, the molecular order decreased, the digestibility of amylose increased, and starches gelatinized	[49]
Yam	Operated at 2450 MHz and full power of 700 W (130–140 °C) for 5 min, then at 100 W for 2 h (60–70 °C)	The moisture content decreased, the total starch content decreased, the amylose content increased, the slow digesting starch content was high, and the swelling power decreased	[71]
*Fritillaria* *Thunbergia*, Miq	Operated at 2450 MHz and full power (700 W) for 5 min (130–140 °C), and then at 100 W for 30 min (60–70°C)	The total starch content was low, the swelling force was reduced, the surface of the grain was not uniform, there were a lot of lamellar bands, the crystal structure was destroyed, and the digestible starch was increased	[72]

The main cause of starch crystallization is attributed to amylopectin. After microwave treatment, the crystallinity of starch molecules generally decreases, which indicates that amylopectin should decrease after treatment. As starch chains fracture, the double helix relaxes and crystallinity decreases facilitating rearrangement of internal structures [64]. In maize starch, however, crystallinity first increased and then decreased, indicating that microwaves first caused the molecular chain to break along crystallization planes, so that crystallinity increased [64]. The breakage of molecular chains also contributes to the degradations of starches to form monosaccharides and enhance acidity [65].

The rearrangement of molecular chains of starch improves the combination between starch and water molecules, causing expansion and gelatinization of the starch easily [14], Gelatinization of starches is usually accompanied by an increase in viscosity. When starch molecules vibrate under the influence of microwave radiation, particles of starch rupture and amylopectin rearranges and viscosity increases [66]. However, the viscosity of potato starch decreased after microwave treatment [70], which we attribute to breaking of intramolecular hydrogen bonds and strengthening of intermolecular hydrogen bonds by microwave radiation. These forces reduce the swelling power of the starch and increases its solubility [72].

As all of these changes occur, the digestibility of the starches increases because the internal structure of the starch has been damaged, surface cracks and deep cavities caused by microwave result in greater contact with amylase [49], and leading to higher digestibility after starch gelatinization [73], however, the slow digestion of yam starch increased after microwave treatment [71]. This behavior is linked to an increase in amylose, which is thought to be more suitable for people with diabetes, while amylose is associated with low digestibility. However, in the chestnut starch experiment, the amylose tended to decrease due to a loss of amylose caused by the breakdown of the amylose chain [69]. The effect of microwave drying on the crystal zone was faster than that in the amorphous zone, and the structural destruction of the amorphous zone makes the starch more sensitive to hydrolase. Therefore, appropriate power levels and times of exposure should be selected according to the digestibility of the starch after treatment by microwaves.

#### Protein

Proteins are important functional substances found in living organisms, and changes in the structure of proteins modify its original biological activity in the human body. Denaturation of proteins destroy their normal physiological functions, damage cells, and cause disease [74]. In the process of heating, the protein is prone to denaturation and produce carcinogens [75]. Several studies have demonstrated that exposure to microwave radiation can cause nerve damage in humans [76], as well as oxidative stress and inflammation [77]. However, microwave radiation appears to have little if any adverse effects on plant proteins; in fact, one study was found that microwave radiation destroyed harmful proteins and improved some properties of proteins [78].

Another study discovered damage to proteins in Lotus seeds after microwave treatments [79]. The effects of exposure to heat and radiation and the subsequent formation of free radicals damage the intramolecular forces, causing gradual exposure of tryptophan, initially trapped in molecules, it breaks free of intramolecular hydrogen bonds causing the orderly arrangement of atoms in proteins to become disordered.

Under the influence of electromagnetic fields, polar radicals in proteins rotate and collide, and the frequency and intensity of interactions between hydrophilic groups on the surface of proteins and the water molecules increase which promotes hydration. At the same time, the destruction of the intramolecular forces in protein molecules promotes aggregation of loose proteins [79]. After heating, the level of thrombin, a protein that can be fatal and is commonly found in legumes, decreased, and the destruction of trypsin inhibitors was greater in wet beans [78]. Therefore, the use of microwave to heating bean seeds, not only reduces toxic proteins found in these seeds, but it improves digestibility. Hydration of proteins also facilitates their emulsification, but aggregation of proteins and exposure to hydrophobic radicals reduce their solubility. The effect of microwaves on secondary structures found in proteins was to increase surface area, thus microwave technology can be used to pretreat proteins through enzymatic hydrolysis [80]. In summary, protein will be denatured after microwave treatment, breaking down harmful proteins effectively, and the loose structure caused by microwave can also improve the digestibility of proteins.

#### Lipids

Lipids are widely found in nature, and most of them exist in seeds, fruits, and animals. Because many phenolic compounds are contained in lipids, they perform a variety of biological functions. Vegetable oils contain a large number of unsaturated fatty acids, which have better antioxidant activity. However, lipids will be oxidized and degraded during heating. Free radicals are produced during microwave heating, which makes lipids more susceptible to oxidation under microwave heating conditions than conventional heating.

Rancidity of cottonseed oil under conditions of microwave heating happens fast, and the content of peroxide and secondary oxidation products, and levels of peroxide in the product increased, and the degree of peroxide reactions positively correlated with power levels. The reaction mechanism of peroxide is related to reactive free radical methylene in lipids produce the free radicals which easily react with oxygen to produce peroxide. Microwave radiation and high temperatures accelerate the formation of these free radicals [81]. Lipid can be degraded to secondary oxidation products after peroxidation, which is the main factor in oil rancidity. During the drying process in microwave heating, the content of phenols decreases, which in turn reduce the oxidation stability of oil [29]. However, microwave-assisted extraction of flaxseed oil had a better effect and showed greater oxidation stability than the Soxhlet extraction, because microwave irradiation damages the internal structure of the sample material, and the rupture of cell structure promotes rapid release of oils from cells. Changes in the structure of cytoderm increased porosity and helped to drive the oil through the cytoderm [82]. The microwave-assisted extraction contained a large proportion of unsaturated fatty acids in the silkworm pupal oil, thus microwave-assisted extraction had no effect on the double bonds in the oil [83], and enzymatic hydrolysis of oil in the palm fruit stopped after microwave treatment [84]. This behavior is attributed to the functional denaturation of proteins by exposure to microwaves. In summary, medicinal materials in oils cannot be stored for long after microwave treatment because of the now accelerated rate of rancidity.

Overall, microwave extraction of oils has a good chance for effective outcomes. It is also believed that microwave can promote the Maillard reaction, and have a positive impact on the shelf life of oil [82]. The deterioration of oil during heating is mainly due to its oxidation reaction. The oil quality was stable in the first 3 min of microwave heating [85]. For example, Moringa oleifera lam. Seeds microwaving for 1 min had better yield and quality than untreated Seeds [86]. In addition, the oil quality can also be improved by adding antioxidants or catalysts, which is mainly for the situation requiring microwave heating for more than 3 min. Solid suspensions such as a mixture of K3PO4 and clinoptilolite or bentonite can absorb microwave energy to produce a protective effect on bio-oil [87].

#### Vitamin

Vitamin is a kind of natural antioxidant which plays an important role in human body and has anti-tumor activity [88]. It also has a positive effect on the prevention of diseases. Vitamins are sensitive to both heat and light [89]. During processing, vitamins are lost due to thermal degradation, photoenzymatic reactions, and oxidation reactions [90]. Vitamin C is a good antioxidant that fights tissue damage caused by free radicals [91]. Compared to traditional method of heating, the vitamin content retained after microwave heating was much higher [20].

Vitamin C in broccoli is degraded by heating under microwave conditions, but the rapid rise in temperature was more conducive to the retention of vitamins as compared to traditional methods of heating. In addition, vitamins are also lost due to leaching [90], while the microwave-assisted drying process is more conducive to the preservation of vitamins. The higher the power level, the larger the content of vitamin C retained [92]. However, Horuz et al. [25] believed that under microwave conditions, vitamin C in tomatoes would degrade due to the electromagnetic force of microwaves, and the temperature rise in the sample caused by microwaves causes thermal damage and drives oxidation reactions, thus increasing the loss of vitamin C. In conclusion, the retention of vitamin C at high microwave power is attributed to periods of time shorter in duration but higher in microwave power. The rapid loss of moisture caused by microwave heating is conducive to the preservation of vitamin C, therefore, when processing vitamin-rich products, microwave heating at high power should be applied for short periods of time. At the same time, water soluble vitamins should be heated as much as possible in a dry environment.

### Secondary metabolite

#### Polyphenols

Polyphenols have strong antioxidant activity because they possess multiple hydroxyl radicals. It has been reported that phenolic compounds have strong scavenging affinity for peroxide-free radicals [93]. Phenols are found in many plants, including proanthocyanidins in grapes, gingerol in dried gingers, and gastrodin in *Gastrodia elata*. During dry storage, the release of antioxidants reduces deterioration caused by oxidation, so the loss of polyphenols should be limited during processing [94].

In microwave-assisted extracts, irradiation could increase the total phenolic content significantly. On one hand, rapid heating induced by microwave radiation caused the plants to release polyphenols to resist external interference such as air oxidation [93]. On the other hand, the combination of microwave energy and moisture can easily penetrate the plant matrix, promote dissolution of polyphenols in cell tissue, and increase solubility [95]. Increasing extraction time and power levels was increased total phenolic content in the extract. However, for heat-sensitive polyphenols, such as proanthocyanidins, higher temperature accelerated decomposition and destruction [96]. When using microwaves in extraction, attention should be paid to the decomposition temperature of the extracted material to avoid damaging the compound by extending the extraction time, and direct extraction of heat-sensitive substances is not suitable for the microwave method.

Compared to traditional drying methods, microwave drying can cause greater damage to tissues and cells in plants, and may cause samples to swell due to microwave-induced dipole rotation and rapid transfer of mass under microwave power. In addition to promoting dissolution of polyphenols, the destruction of cells and tissues increased the area of contact between polyphenols and oxidase, which promotes oxidation of polyphenols [97]. However, some studies showed that the content of gingerol in ginger dried using microwave radiation first increased, then decreased, and the DPPH scavenging activity was high [98]. These results suggest that the microwaves did not damage the gingerol in the early stages, and there were other antioxidant compounds produced or dissolved in the ginger in latter stages. When use of microwave radiation was introduced under vacuum conditions, degradation of polyphenols was reduced [99]. At the same time, rapid heating destroys the oxidase in the products and inhibit the enzymatic browning to increase the substrate of phenol and resisting the oxidation of polyphenol in the drying process [100]. Meanwhile, less oxygen content in a vacuum environment reduces oxidation of polyphenols. And the inhibition of browning can improve the color of the sample, making it more colorful. Microwave-dried green tea has high content of catechins and bright color [101].

**Fig. 6:**
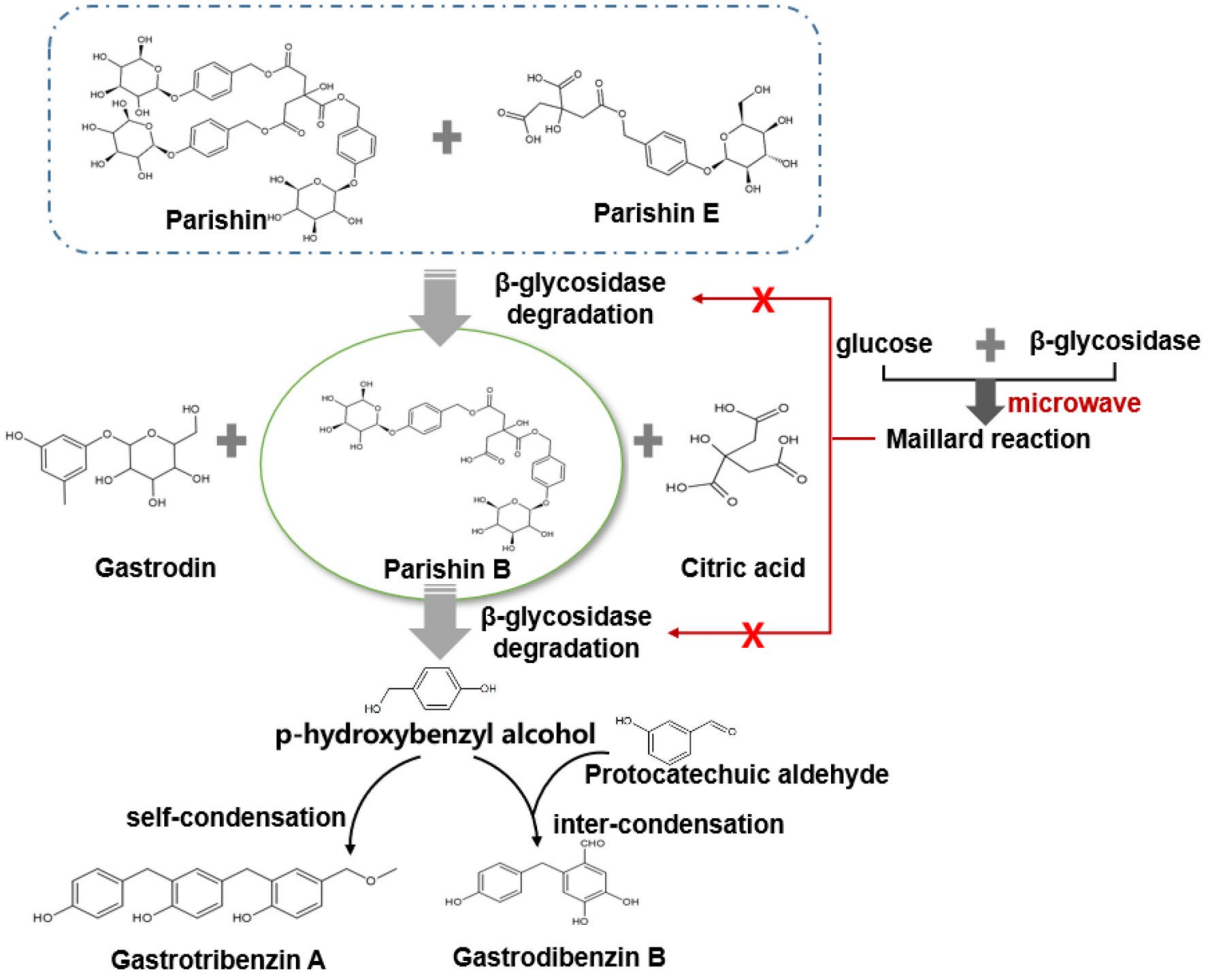
Changes of phenolic substances in *Gastrodia elata* under heating condition

Gastrodin in *Gastrodia elata* Blume is a phenolic glycoside. Free gastrodin and benzyl alcohol form when ether bonds or ester bonds are broken in the heating process. Changes of phenolic substances in *Gastrodia elata* under heating conditions are shown in Fig. 6 [102]. High temperatures can destroy the enzyme gastrodin and inhibit enzymatic degradation by gastrodin [103]. However, most compounds of *Gastrodia elata* Blume are sensitive to heat, so the initial processing time of *Gastrodia elata* should be as short as possible to minimize the loss [104]. Characterized by selective heating and porosity materials of microwave radiation can promote the release of phenolic glycosides, and destroy β-D-glucosidase, thus preventing the hydrolysis of gastrodin [105]. Therefore, microwave drying can achieve “enzyme inactivation to preserve glycosides”, but the temperature and duration required to dry plant tissue using microwave radiation must be controlled to reduce degradation of compounds at high temperatures.

#### Alkaloids

**Fig. 7:**
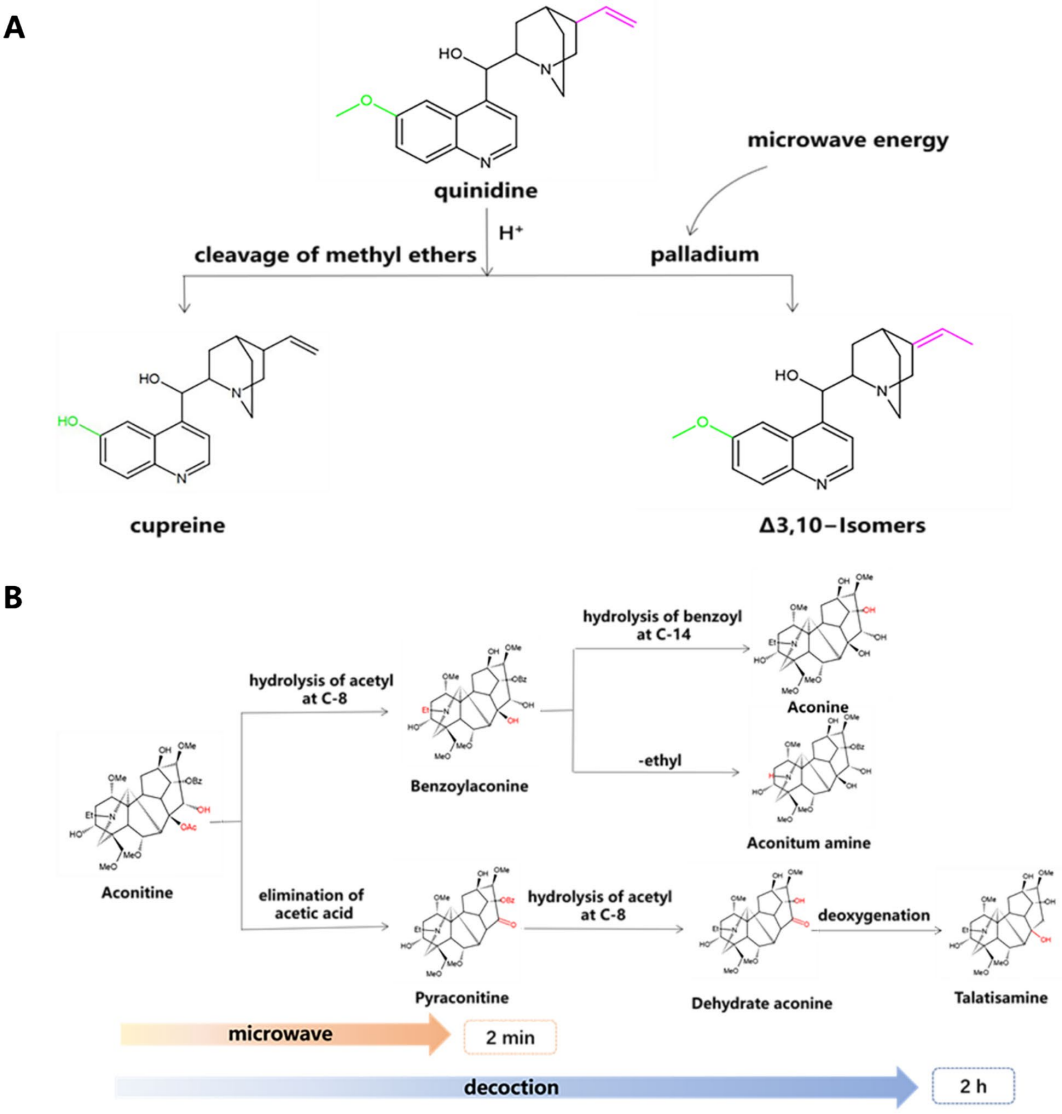
**A** Isomerization of cinchona alkaloids. **B** Conversion of aconitine

Through the study of *Fritillaria thunbergii Miq* [72]*.* and *Chelidonium majus*. [106], it was found a decrease in the content of peimine after microwave drying, attributed to high temperatures. After microwave extraction, the alkaloids in greater celandine contained the greatest content. Compared to traditional methods such as crushing, soaking extraction and modern methods such as supercritical fluid extraction, we conclude that treatment with microwave radiation is the only way to detect the berberine, so the use of microwave extraction demonstrated superior antibacterial ability. Lipińska et al. [107] found that the high energy provided by microwave radiation promoted formation of the transition state in alkaloid isomerization which in turn accelerated structural changes. Under microwave catalysis, the site of isomerization reactions is more selective, resulting in higher purity of product (Fig. 7A). Microwaves work on double bonds by rearranging the electron clouds and promoting isomerization. When the structure of alkaloids changes, their properties change. Microwave treatment reduces toxicity in medicinal materials containing toxic alkaloids. For example, toxic components such as aconitine were hydrolyzed and disappeared shortly after treatment [108]. Traditional decoctions at high temperature takes two hours to complete detoxification, and undetected toxic substances likely remain [109].

After treatment, the content of aconitine decreased and the content of benzoyl aconitine and aconine increased, indicating that the diester-diterpenoid alkaloids were transformed into monoester-diterpenoid alkaloids and amine-diterpenoid alkaloids (Fig. 7B [110]). For toxic components such as yunaconitine [111], microwaves are thought to destroy the ester bonds in these compounds thereby enhancing efficacy and reducing toxicity.

#### Flavonoids

Flavonoids help regulate biological activities through antioxidant, anti-tumor, antibacterial and hypoglycemic properties. They are bioactive component in many kinds of medicinal materials, fruits and vegetables [112, 113], therefore, the degradation of flavonoids should be limited, and stability should be improved during the processing of products whenever possible. However, under the condition of heating, flavonoids will degrade and their antioxidant activity will be reduced. In the study of the effects of microwave heating on flavonoids, it was found that in addition to the influence of microwave power and heating time in microwave-assisted drying, the structure of the flavonoids also affects degradation rates, while the increase in hydroxyl radicals promotes degradation of flavonoids [114]; therefore, flavanols are prone to degradation. The biological activity of flavonoids depends on their structure, the number of hydroxyl radicals and functional groups [115]. Flavanols are more bioactive than flavonoids, and the degradation of these compounds should be avoided during drying.

Many flavonoids are degraded during processing, but only myricetin and rhamnetin showed slight decomposition in ears of corn in the first 5 min under 500 W microwave radiation. As exposure time increased, the content of myricetin, kaempferol, quercetin, and rhamnetin decreased, while degradation of rutin and naringin was minimal [114]. Yields of hyperin and vitexin in hawthorn after extraction were higher [116], and the results indicate that glycoside derivatives are more stable than aglycones when irradiated by microwaves.

The loss of flavonoids in material dried by exposure to microwaves is greatly reduced compared to conventional methods of drying, and the retention rate for anthocyanins in sweet potato was higher after microwave treatment than that retained in traditional drying methods [117]. The degradation of quercetin derivatives in apple slices using microwave radiation in a vacuum to dry them was lower than expected, and the degree of yellowing was higher than that achieved in air drying.

Characteristics of microwave treatments of short duration at low temperatures caused only minor changes in color. The degradation of the quercetin derivatives in the samples that were air dried and pretreated in a microwave vacuum chamber were reduced [118]. This may be due to the inactivation of degrading enzymes during pretreatment and the reduction of oxidative degradation in anoxic environment. However, quercetin and its derivatives will aggregate in fruits to form polymers. The polymers will depolymerize and be converted to basic units under the effect of microwave radiation [119]. After depolymerization, monomer compounds are easily destroyed and may be converted to other flavonoids. For example, a flavonol-like quercetin may be converted to catechin and epicatechin. In the process of drying by microwave, the parent nucleus structure of flavonoids was retained, and the destruction of hydroxyl radicals in flavanols declined, while the biological activity of flavonoids was maintained. To avoid damaging of flavonoids after microwave heating, microwave radiation can also be considered as a pretreatment step.

#### Organic acids

Natural organic acids are common in many plants, and contribute to the sensory properties of the plants; they also affect antioxidant activity. The antioxidant activity of organic acids is derived from chelation with metals [120]. In the process of extraction, hydrolysis of organic acids caused by microwave radiation increased the binding sites in organic acids, improving the antioxidant activity of organic acids.

When cooked with heat, The organic acids found in plants, such as oxalic acid, citric acid, malic acid will be lost significantly. Galega Kale cooked in a microwave oven contains more oxalic acid than kale cooked in a vacuum or using traditional method [120]. However, human ingestion of oxalic acid reduces calcium absorption [121], and food with high oxalate content may cause the recurrence of kidney stones [122]. Therefore, in addition to better retention of nutrients in the samples, the elimination of oxalic acid form samples during processing should also be considered. For plants rich in oxalic acid, methods such as stewing can be adopted to minimize the oxalic acid content.

The chemical structure of natural organic acids changes after exposure to microwave radiation. After blanching with the method, the content of gallic acid in fruit peelings increased, the content of caffeic acid increased and then decreased, and the content of ferulic acid decreased [123]. The increase of gallic acid and caffeic acid in the early stages may be related to hydrolysis of ester compounds, while the decrease in ferulic acid and caffeic acid may be caused by the destruction of double bonds during radiation. Microwave-assisted extraction of ellagic acid showed that microwave hydrolyzed ellagic acid rapidly to gallic acid [124]. This is attributed to the rapid hydrolysis of ester polymers induced by exposure to microwaves. The antioxidant activity of clove increased after treatment, and the tannin content in the microwaved extract was high [125]. The main metabolites of tannic acid degradation were gallic acid and glucose [126], therefore, the increase in antioxidant activity in cloves was not only due to the hydroxyl radicals in tannic acid, but also to the breaking of ester bonds in tannic acid to form reducing sugars. As for organic acids with few double bonds and linked sugar radicals to start with, microwave treatments can further reduce their molecular weight to promote absorption. After ester bonds fracture, the content of hydroxyl radicals and reducing sugar increased which enhances the antioxidant properties of organic acids.

#### Saponins

Saponins, plants in the family Araliaceae such as Panax ginseng, and Panax notoginseng have great antibacterial properties. Its activity is related to the number and structure of the monosaccharides in the ligands of the saponin chain, which is key to its antibacterial activity [127]. A glycosidic bond is a polar bond, thus microwave-induced dipole rotation can increase the reactivity of glycosidic bonds and make them easy to break [128]. Therefore, microwave treatment may be detrimental to the antibacterial activity of saponins. Membranolytic activity in saponins is related to the interaction between saponins and cell membranes, and the toxic effect of saponins on cell membranes contributes its antibacterial properties. Some studies have shown that the membranolytic activity in saponins increases sharply after hydrolysis [129]. Microwave radiation also has a selective heating effect on ester bonds. Therefore, to avoid the destruction of antibacterial activity in saponins by microwaves, the conditions required for selective hydrolysis of ester bonds should be explored.

**Fig. 8:**
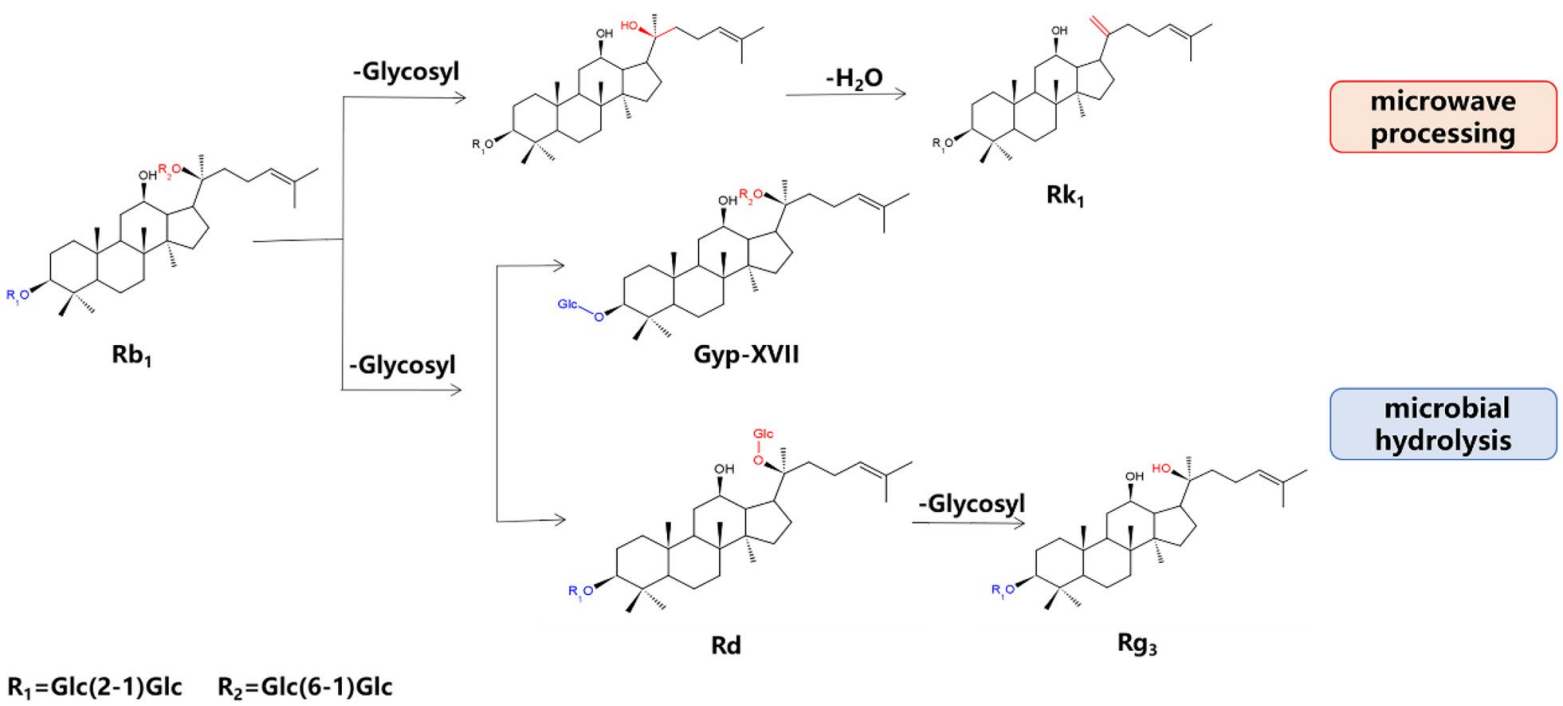
Changes in ginsenoside

The main saponins in raw Panax notoginseng demonstrate hemostatic activity. After microwave treatment, the main components in Panax notoginseng were transformed, 20-Rg3 was generated by the breaking of Rb1 glycosidic bonds, and Rh4 was generated by Rg1; together. These three mechanisms comprise the components of Panax notoginseng and control hemostatic activity after treatment [128]. Compared to other methods, microwave processing produces Rg3 more quickly and reduces generation of unwanted by-products. The changes in ginsenoside are shown in Fig. 8. The microwave-processed Panax notoginseng had stronger blood-enriching activity and hemostatic activity. Although the heating rate of microwave heating was faster, the degradation rate of saponins caused by it was the same as that caused by conventional heating [130]. Therefore, microwave treatment did not affect the storage of saponin-rich medicinal materials. However, when using microwave heating, attention should be paid to the control of its conditions. If the reaction stays in the intermediate step, or excessive hydrolysis is not conducive to the development of medicinal herbs.

#### Terpenoids

Most terpenoids have the structure of conjugated diene, so they have strong antioxidant capacity. During the heating process, the double bonds in terpenoids are easily oxidized or isomerized and degraded. Carotenoids are fat-soluble pigments that give plants color like yellows and reds. Changes in the color of plants signal a loss of terpenoids. The color of carotenoids disappears after oxidation; only differences in saturation remain if isomerization occurs [131]. Microwave-assisted extraction is a unique method and considered the most suitable method for extraction of thermostable terpenoid pigments, such as carotenoids. The heat generated by exposure to microwaves can be focused on the sample without heating the container, thus shortening extraction times [132].

Protection of terpenoids can preserve the color and odor of products during microwave-assisted drying. Chen et al. [133] found that crocin—the active ingredient in saffron—was well preserved after microwave drying. Through color comparisons, saffron acquired a high degree of browning with microwave drying, which is attributed to the high temperatures generated.

Aromatic compounds such as safranal were well retained after microwave treatment, and drying using infrared frequencies produced similar effects, presumably because both the heat is generated by the action of are electromagnetic waves on plant tissue. After microwave drying at high levels of power, the aroma of ginger becomes stronger and colors intensify, while the main volatile components such as zingiberene and curcumin are significantly reduced; however, retention of volatiles was still superior to that in food dried using a conventional method such as an electric oven [134].

The proportion of linalool in basil decreased sharply after microwave drying [135], probably due to volatile substances leached from the surface. By observing the structural transformation of astaxanthin, a carotenoid substance, it was found that trans-astaxanthin transforms into cis structure after absorbing microwave energy [136]. Therefore, the destruction of plant cells and tissues by microwave radiation accelerates dissolution of terpenoids, and leaches volatile terpenoids from some samples can leave a pleasant fragrance, but heat-sensitive components are easily oxidized and degraded in the heating process, but the process can be controlled using a combination of microwave radiation and a vacuum. When the cis−trans structure of the substance is required, attention should focus on control of the microwave conditions.

During microwave treatment, the transformation of natural product composition will change the quality and activity of the natural product. Inactivation of enzymes and retention of pigments such as anthocyanins after microwave treatment preserve the color of products [24, 137]. In microwave-treated peanut oil, the acid and peroxide values are increased, but a new composition, pyrazines, gives the oil a better flavor [138]. In addition, protection of polysaccharides, polyphenols, vitamins and other metabolites makes the natural products processed by microwave have better biological activity, such as antioxidant, anti-free radical and antibacterial activities [139]. However, in the application of microwave in the food field, it has been found that microwave processing may cause starch to produce toxic substances or structural changes to expose more potential toxic sites [140]. But such situation has not been reported in the field of medicinal materials processing. Therefore, further attention should be paid to the safety of microwave.

## Modern application of microwave technology in pharmaceutical field

In recent years, microwave technology has attracted more and more attention in the pharmaceutical field due to its unique advantages, especially in the field of Chinese medicine processing. At present, it is mainly used in the synthesis of composite materials, chemical structure modification, as well as the drying, extraction, sterilization of Chinese medicinal materials. At the same time, it is also used in the treatment of traditional Chinese medicine wastewater.

## Drying of Chinese medicinal materials and preparations

Compared with traditional Chinese medicine drying methods, microwave technology not only has the characteristics of convenient operation, heat rapidly, good product quality and low energy consumption, but also has excellent sterilization effect while drying. It is found that microwave drying technology can be used for the processing of Chinese medicinal materials which are rich in volatile oil, polysaccharides or saponins. For example, microwave drying can effectively reduce the loss of ferulic acid in Angelica sinensis [141], increase the content of monomer saponins in ginseng. At present, microwave technology is widely used in robinia locust, Salvia miltiorrhiza, Chinese yam and other medicinal materials drying [142]. It has also been studied in the drying of ginseng, scutellaria baicalensis, rehmannia rehmanniae and scrophulariae radix. Microwave drying technology can also be used to dry the extract of traditional Chinese medicine, especially the extract with high sugar content. It takes about 1–2 days to dry the extract of traditional Chinese medicine through conventional drying methods, while the microwave drying technology can effectively shorten the drying time. According to the properties of the extract, it takes about 2–4 h to realize the complete drying of the extract. At the same time, microwave drying technology can also be used to dry some Chinese medicine preparations, such as Liuwei Dihuang pill and Ma Ren pill. It is worth noting that after microwave drying, some Chinese medicine pills which are rich in oil may appear unqualified dissolution and dispersion time limit. It could be because after microwave drying, oil components overflow to fill the position of water loss and increase the hydrophobic of the pills, leading to the unqualified dissolution and dispersion time limit.

### Sterilization of Chinese medicinal materials and preparations

Microwave sterilization technology, as a new sterilization technology, not only has a variety of sterilization mechanisms, but also has its unique advantages. Microwave technology for the sterilization of traditional Chinese medicine and its preparations can be carried out at low temperature (60–80 ℃). And it has strong penetration, can make the material inside and outside concurrent heating, heating evenly, to achieve all-round sterilization. At the same time, microwave technology sterilization speed, and can be carried out at the same time with the material drying, without other heating steam and pipeline auxiliary facilities, high efficiency and energy saving. In addition, microwave technology sterilization can be carried out at the same time as material drying, without other heating steam and pipeline auxiliary facilities, to achieve high efficiency and energy saving. At present, microwave technology has been used in the sterilization of Chinese medicinal materials and their pills, powders, oral liquid as well as eye drops [143]. For example, after microwave sterilization, the bacteria, mold, yeast and escherichia coli in the decoction pieces of Euphorba officinalba were significantly reduced. After powder preservation, the bacteria and mold contained in the decoction pieces were all in line with the corresponding microbial limit [144]. There are some scholars compared the components of preparation before and after microwave sterilization, and found that the active components of Snagan-Chuanfritillaria powder were stable, and the transfer rate of Fritillaria alkali was more than 94%, indicating that microwave sterilization process is stable and reliable [145].

### Extraction of active ingredients

Microwave-assisted extraction technology, also known as microwave extraction, refers to the method and technology of extracting the chemical components of natural medicinal minerals, plants and animal tissues by using appropriate solvents in a microwave reactor. Compared with the traditional extraction method, microwave-assisted extraction technology can not only reduce the use of solvent, the waste produced and energy consumption, shorten the extraction time, but also improve the extraction rate and purity, reduce the production cost. At present, microwave extraction can be used for the extraction of alkaloids, polysaccharides, volatile oil, brass and organic acids and other components. It is worth noting that microwave technology can only be used for the extraction of heat stable components. For heat sensitive components, such as polypeptides, proteins and enzymes, denaturation and inactivation may occur. However, in recent years, microwave technology has been combined with other technologies, such as decompression extraction and ultrasonic extraction, which can realize the rapid extraction of active components at low temperature, reduce the damage to the components, and can maintain the original form of natural product to a large extent.

### Treatment of Chinese medicine wastewater

Microwave has strong penetration, can heat reaction system quickly and evenly, has a catalytic effect. In recent years, it has been applied in the field of water treatment. Traditional Chinese medicine wastewater contains lignocellulose, saponin and other substances that are difficult to be biodegradable. It has high chemical oxygen content and chromaticity, so it is difficult to treat. There are many problems in traditional physico-chemical and biological treatment methods, such as large consumption of reagents, large scale of treatment facilities and secondary treatment of sludge. Research shows that the microwave assisted Fenton system to treat traditional Chinese medicine wastewater, the degradation efficiency of chemical oxygen content and chromaticity is high, the reaction time is significantly shortened, H_2_O_2_ utilization efficiency is significantly improved, to meet the emission standards [146].

## Safety analysis of microwave technology in pharmaceutical field

The safety of microwave technology has always been a concern of people. Different from the traditional heating method, microwave makes the polar molecules in the material vibrate at high frequency and generate heat by rapid friction. The whole reaction system is more violent, and there are more uncertainties in the production, leading to potential health risks. In the field of food, studies have found that microwave processing may produce toxic substances in food starch, or make the structure change to expose some more potentially toxic sites [147]. For example, microwave heating will increase the content of α -dicarbonyl compounds, which lead to an increase in harmful glycosylation products [148]. And because of the oxidation effect of microwave, microwave cooking has higher levels of cholesterol oxides than other cooking methods, which can also have negative effects on human health [149].

Different from the food field, the pharmaceutical field pays more attention to whether the quality and efficacy of drugs will be changed after the treatment of microwave technology, and whether the change can be controlled, especially in the processing of Chinese medicinal materials. Unlike chemical medicine, composition of Chinese medicinal materials is complicated. There are some uncertainties in the changes of composition under microwave treatment. In practice, the interaction between microwave processing technology and the safety and pharmacodynamic value of medicinal materials should be fully considered. Furthermore, scientific and reasonable processing strategies and processes should be selected for different components to ensure the effective application of advanced microwave technology and reduce the loss of components and safety hazards caused by microwave processing. Meanwhile, we should continue to learn and draw on the advanced experience in the food field, strengthen the research on the safety of microwave technology in the pharmaceutical field, establish a standard processing mode, strengthen the process research under the conditions of industrial microwave, and reduce the uncertainty and potential risk of microwave technology.

## Conclusions

At the present time, as a new technology, microwave technology has been extended from food drying to medicine, materials, chemistry and other fields. Microwave technology has the advantages of: (1) fast heating speed, ensure the quality of natural products is stable; (2) realize sterilization while heating, and avoid chemical pollution; (3) selective effect of polar bonds, local heating; (4) Damaging the cell structure of natural products, promoting the dissolution of effective ingredients. Based on these advantages, microwave technology can ensure the efficacy of medicinal materials, is a new technology that can be promoted in the field of medicinal materials processing. It has great significance to the preservation, dehydration, drying of plant medicine. Transforming the composition of natural products by microwave radiation is a promising method, which can improve the efficiency constituent and reduce the toxicity at the same time of drying and extraction, so as to increase the safety and effectiveness of natural products. In addition, the selectivity of microwave technology makes it very promising in synthesis catalysis.

However, the use of microwave technology is still inadequate: (1) excessive microwave heating rate leads to the destruction of the internal structure of the sample; (2) rapid water loss causes the reaction to stay in an intermediate stage; (3) there are many influencing factors and the process is not easy to control. Therefore, when microwave technology is used for drying, it is often used in combination with other drying technologies, such as microwave vacuum drying, microwave freeze drying, microwave hot air drying and microwave fluidized bed drying, so as to make the sample uniform and fast drying [150, 151]. And in the use of microwave technology, it is necessary to consider its possible negative effects. For example, the original non-toxic potato starch will produce toxic acrylamide after high power microwave treatment [147]. These possible toxic substances should be taken into account. Therefore, more attention should be paid to the changes of natural products caused by microwave in the processing of natural products, so as to realize the application of microwave technology “to benefit and avoid harm”.

## Data Availability

Not applicable.
